# Egocentric vision-based detection of surfaces: towards context-aware free-living digital biomarkers for gait and fall risk assessment

**DOI:** 10.1186/s12984-022-01022-6

**Published:** 2022-07-22

**Authors:** Mina Nouredanesh, Alan Godfrey, Dylan Powell, James Tung

**Affiliations:** 1 Department of Mechanical and Mechatronics Engineering, University of Waterloo, 200 University Ave. W, N2L 3G1 Waterloo, Canada; 2 School of Rehabilitation Science, Faculty of Health Sciences, McMaster Univerdity, 1400 Main Street West, L8S 1C7 Hamilton, ON Canada; 3 Department of Computer & Information Sciences, Northumbria University, 2 Ellison Pl, NE1 8ST Newcastle upon Tyne, UK

**Keywords:** Free-living digital biomarkers, Egocentric vision, Free-living gait analysis, Wearable sensors, Terrain type identification, Deep convolutional neural networks

## Abstract

**Background:**

Falls in older adults are a critical public health problem. As a means to assess fall risks, free-living digital biomarkers (FLDBs), including spatiotemporal gait measures, drawn from wearable inertial measurement unit (IMU) data have been investigated to identify those at high risk. Although gait-related FLDBs can be impacted by intrinsic (e.g., gait impairment) and/or environmental (e.g., walking surfaces) factors, their respective impacts have not been differentiated by the majority of free-living fall risk assessment methods. This may lead to the ambiguous interpretation of the subsequent FLDBs, and therefore, less precise intervention strategies to prevent falls.

**Methods:**

With the aim of improving the interpretability of gait-related FLDBs and investigating the impact of environment on older adults’ gait, a vision-based framework was proposed to automatically detect the most common level walking surfaces. Using a belt-mounted camera and IMUs worn by fallers and non-fallers (mean age 73.6 yrs), a unique dataset (i.e., Multimodal Ambulatory Gait and Fall Risk Assessment in the Wild (MAGFRA-W)) was acquired. The frames and image patches attributed to nine participants’ gait were annotated: (a) outdoor terrains: pavement (asphalt, cement, outdoor bricks/tiles), gravel, grass/foliage, soil, snow/slush; and (b) indoor terrains: high-friction materials (e.g., carpet, laminated floor), wood, and tiles. A series of ConvNets were developed: *EgoPlaceNet* categorizes frames into indoor and outdoor; and *EgoTerrainNet* (with outdoor and indoor versions) detects the enclosed terrain type in patches. To improve the framework’s generalizability, an independent training dataset with 9,424 samples was curated from different databases including GTOS and MINC-2500, and used for pretrained models’ (e.g., MobileNetV2) fine-tuning.

**Results:**

*EgoPlaceNet* detected outdoor and indoor scenes in MAGFRA-W with 97.36$\%$ and 95.59$\%$ (leave-one-subject-out) accuracies, respectively. *EgoTerrainNet*-Indoor and -Outdoor achieved high detection accuracies for pavement (87.63$\%$), foliage (91.24$\%$), gravel (95.12$\%$), and high-friction materials (95.02$\%$), which indicate the models’ high generalizabiliy.

**Conclusions:**

Encouraging results suggest that the integration of wearable cameras and deep learning approaches can provide objective contextual information in an automated manner, towards context-aware FLDBs for gait and fall risk assessment in the wild.

**Supplementary Information:**

The online version contains supplementary material available at 10.1186/s12984-022-01022-6.

## Background

Falls in older adults (OAs, $>65$ yrs) are one of the most important public health problems worldwide, which impact one in three OAs at least once each year [1]. OAs’ falls have a multifactorial etiology [2] with risk factors generally categorized as intrinsic/biological (e.g., gait and balance impairment, visual disorders) and extrinsic/environmental (e.g., irregular or slippery surfaces). Gait and balance disorders and environmental hazards have been reported to be the most important risk factors contributing to $\approx 17\%$ and $\approx 31\%$ of falls in OAs, respectively [3]. To assess the exposure to risk factors, fall risk assessment (FRA) methods have been developed, which informs selection and timing of interventions to prevent fall incidents. Commonly used clinician-administered tests in controlled conditions (e.g., Timed Up and Go [4]) can provide valuable insights on specific aspects of an OA’s intrinsic risk factors at discrete points in time. However, these in-lab/in-clinic approaches have exhibited a low-to-moderate performance in the identification of fall-prone individuals [5]. To address this limitation, recent attention has been focused on free-living FRAs using wearable inertial measurement units (IMUs) to assess OAs’ activities in their natural environments. Proposed free-living FRA approaches (e.g., 24 studies reviewed in [6]) have investigated relationships between falls and IMU-derived free-living digital biomarkers (FLDBs), primarily extracted from gait bouts [6]. Gait-related FLDBs include macro (e.g., quantity of daily: steps [7], missteps [8], and turns [9]) and micro (e.g., step asymmetry [7]) measures. Although these measures can be impacted by both intrinsic and environmental features [10–12], their respective impacts on FLDBs’ fall predictive powers have not been differentiated [6]. For instance, higher variability in acceleration signal (measured by the amplitude of the dominant frequency in the mediolateral direction, as a FLDB) during gait could indicate appropriate adaptation to the environment [13] (and potentially a lower risk of falls) and/or exhibit gait impairment (and potentially a higher risk of falls) [14]. Similarly, frequent missteps (as a FLDB) detected in free-living IMU data can be an indicator of impaired dynamic balance control (and a higher risk for falls [8]) and/or false alarms generated by anticipatory locomotion adjustment while walking on an irregular terrain (e.g., construction site) [15]. This ambiguity in interpretation leads to less precise intervention strategies to prevent falls.

A *context-aware* free-living FRA would elucidate the interplay between intrinsic and environmental risk factors and clarifies their respective impacts on fall predictive powers of FLDBs. This would subsequently enable clinicians to target *more specific* intervention strategies including environmental modification (e.g., securing carpets and eliminating tripping hazards) and/or rehabilitation interventions (e.g., training to negotiate stairs and transitions). Ideally, a context-aware free-living FRA method would be capable of examining the relationships between the frequency of falls, FLDBs, and different environmental fall-related features such as presence of dynamic obstacles (e.g., pedestrians, pets), unstable furniture, lighting condition, and terrain types. As a step towards this longer-term goal, the focus of the present study is to develop an automated method to differentiate between different walking surfaces commonly observed in everyday environments.

A wrist-mounted voice recorder was previously utilized to capture contextual information following misstep events (trips) [16], which could be limited to observations made by the user and may lack spatial and temporal resolution. To objectively identify terrain types, several studies examined the feasibility of using wearable IMU data recorded during gait [17–19]. For instance, machine learning models achieved 89$\%$ accuracy (10-fold cross-validation) to detect six different terrains including soil and concrete using two IMUs in [17]. These studies investigated datasets mostly sampled from young participants in controlled conditions (i.e., walking repetitively over a few surface types with constant properties), and primarily reported machine learning models’ holdout or k-fold cross-validation measures. However, cross-validation approaches such as leave-one-subject-out (LOSO) or models’ assessment using independent test and training datasets represent a more reliable picture of models’ robustness against inter-participant differences and generalizability to unseen data [20, 21]. Additional file 1: Preliminary results for IMU-based surface type identification reports the drastic difference between the k-fold and LOSO results of machine learning models implemented using the same IMU data (an open access dataset [22]) to differentiate between the walking patterns over stairs, gravel, grass, and flat/even surfaces.

Egocentric or first person vision (FPV) data recorded by wearable cameras affords the ability to provide rich contextual information more readily than IMU-based data alone. Additionally, while third-person vision data captured by ambient cameras (e.g., Microsoft Kinect) could provide valuable contextual information in an unobtrusive manner, they are restricted to fixed areas and can be challenged by multiple residents with similar characteristics [23]. In contrast, FPV data can be recorded in any environment with which the camera wearer is interacting, including outdoors [21]. In [24], seven days of data were collected from fallers and controls during daily activities using ankle-mounted IMUs and a neck-mounted camera. Subsequently, the frames attributed to walking bouts were investigated and annotated manually. The most frequent terrain type manually identified for all participants were outdoors on pavement, indoors on carpet and polished or hardwood flooring. Other terrain observations included grass, gravel, and multiple environments. However, the manual identification of walking surfaces, especially in large-scale free-living studies, is a laborious and inefficient process. To advance the field of free-living FRA and gait assessment, there exists a need to develop automated vision-based methods for terrain type specification.

Automated vision-based methods for terrain type identification have been investigated in other fields of assistive technology and robotics (mostly focused on outdoor terrain types [25–27]). For instance, in [28] head-mounted camera data were used for adaptive control of legged (humanoid) robot’s posture and dynamic stability on different terrains. Engineered features such as intensity level distribution, complex wavelet transform, and local binary pattern were extracted and a support vector machine model was developed to categorize 1,000 training images to three classes: (a) hard (e.g., tarmac, bricks, rough metal); (b) soft (e.g., grass, soil, gravels, snow, mud); and (c) unwalkable (static and moving obstructions). Although useful, this approach may not provide sufficient descriptive information to inform FRA. For instance, while snow, gravel and grass were considered into the same class, they would be expected to induce different patterns of gait. A relatively high accuracy of 82$\%$ was achieved when the model was applied to a 40-second video. However, this approach’s high computational cost was considered a limitation. Elsewhere, to control a powered prosthetic leg, a camera and IMU were mounted on the prosthetic and the relationship between image sharpness and acceleration was considered to trigger the camera [29]. Twenty minutes of data were collected from 5 able-bodied participants walking over 6 different types of terrain (asphalt, carpet, cobblestone, grass, mulch, and tile). Using a bag of word approach (SURF), an average classification accuracy of 86$\%$ was achieved based on 5-fold cross-validation. Deep learning approaches have shown strong potential to outperform engineered and bag-of-word-based approaches from many aspects, particularly inference time and accuracy [30, 31]. By integrating both order-less texture details and local spatial information, a Deep Encoding Pooling Network model was developed [32]. The model was trained on the images in Ground Terrain in Outdoor Scenes (GTOS) dataset [27], and tested on GTOS-mobile dataset. The former contains 30,000 images across 40 outdoor terrain classes captured by a camera mounted on a mobile exploration robot with a fixed distance between the camera and the ground. GTOS-mobile data was captured by a mobile phone and with more flexible viewpoint, still relatively close to the ground. Although promising results were achieved, due to low intra-class diversity, limited viewpoint, and restriction to outdoor terrains, the GTOS(-mobile) models may not be generalizable to address the problem of terrain identification in complex everyday environments. More relevant to the context of FRA, data of a chest-mounted camera and Gabor Barcodes [33] were used to automatically detect 17 environmental fall-related features such as slope changes (e.g., ramps) and surfaces (e.g., gravel, grass). Although high (88.5$\%$) accuracy was achieved, the incorporated dataset was restricted to young adults, limited to public environments lacking at-home data. Moreover, only k-fold cross-validation results were reported.

**Fig. 1:**
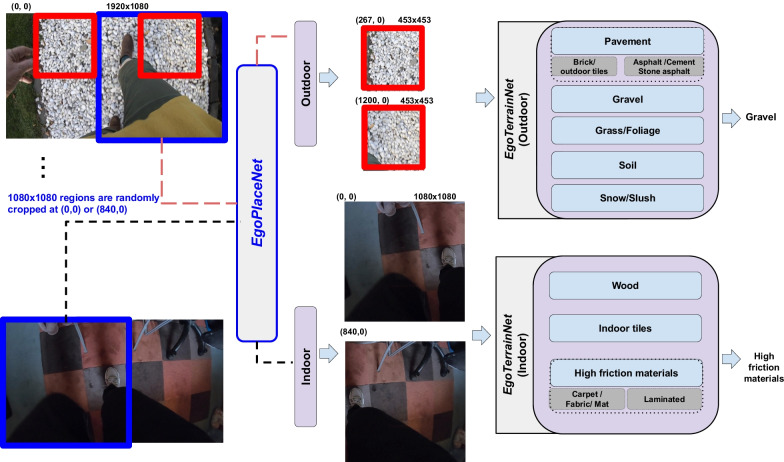
The proposed framework consists of two models: **a** *EgoPlaceNet*, which classifies scenes (one $1080\times 1080$ region for each frame cropped randomly either from right or left corner, the blue square) into indoor and outdoor, and **b** *EgoTerrainNet*, with Indoor and Outdoor versions, which classifies two 453$\times$453 (red squares) and 1080$\times$1080 patches based on the enclosed terrain type

To address the previous research works’ limitations, this paper employs a unique dataset, i.e., Multimodal Ambulatory Gait and Fall Risk Assessment in the Wild (MAGFRA-W), collected from older non-fallers and fallers in out-of-lab conditions and presents a vision-based deep framework to classify level walking surfaces (see Fig. 1). To *maximize* the framework’s generalizability and *minimize* its dependence on sample size, an independent training dataset with *high* intra-class variance was formed by curating data from relevant datasets, such as GTOS ("Assessing and augmenting models' generalizability" Section). The curated dataset includes the following 8 classes (a) outdoor: pavement, grass/foliage, gravel, soil, and snow/slush and (b) indoor: high-friction materials, tiles, wood flooring. Subsequently, the framework’s generalizability to OA’s data and its robustness against inter-participant differences were assessed (e.g., using LOSO cross-validation). The proposed framework provides one of the first investigations into the contextualization of free-living gait and fall risk assessment in OAs.

## Materials and methods

### Recruitment and data collection

The project received ethics approval (reference number: 17589, approval date: 4-Oct-2019) from Northumbria University Research Ethics Committee, Newcastle upon Tyne, UK. All participants gave written informed consent before participating in the study.

**Table 1:**
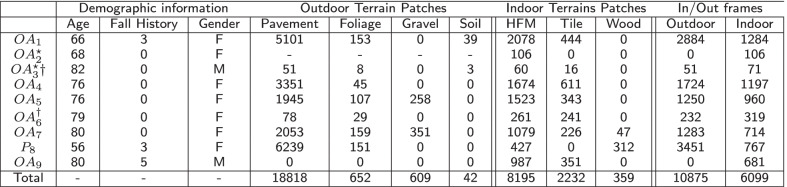
Demographic information and the distribution of crops/frames over different classes

From MAGFRA-W, only frames and patches attributed to walking bouts ≥3steps (level walking) were annotated. Fall history: number of falls in the previous one year, $\star$: camera was unintentionally mounted upside-down by the participants or was set to take photos (not videos) resulted in smaller sample size, †: Participants living in the same home. HFM: high-friction materials

Using wearable IMUs, cameras, and a motion capture system, a unique dataset, Multimodal Ambulatory Gait and Fall Risk Assessment (MAGFRA), was collected from fallers and older non-fallers in laboratory/clinic (MAGFRA-C) and/or *in the wild* (MAGFRA-W) [34]. In the present study, FPV data from nine participants (2 males, 7 females, mean age $\approx$73.6 yrs, 3 fallers) from MAGFRA-W were used (Table 1). One participant’s age was below 65 yrs, but as she was a recurrent faller, her data were considered for further analysis (marked by P8). The number of self-reported falls in the previous 12 months is reported in Table 1.

Considering our previous findings [21, 35], we hypothesized that a waist-level camera would offer a greater resolution of the feet and texture of surfaces than views higher on the body (e.g., a chest-level camera) for the purpose of informing free-living FRA. Moreover, as discussed in our previous research work, waist level views offer a consistent view of the feet even during sharp turns [21]. In contrast, head- and leg-mounted views tend to rotate in anticipation of turns or shift in attention, which reduces views of the feet and the terrain underneath and increases risk of motion blur [28, 29]. Thus, video data were collected using a GoPro Hero 5 Session or Hero 6 Black camera (30fps, 1920$\times$1080, wide view, except for OA2 and OA3 as marked by $\star$ in tables, see "Preprocessing"), centered at each OAs’ waist by means of a belt attachment. The camera was set up to capture top-down views of feet and the regions around them, with no calibration or a strictly reproducible placement procedure on camera’s angle with respect to the frontal plane.

Data collection was performed in (a) public environments within Northumbria University, during which participants had to navigate through different indoor and/or outdoor environments while walking alongside a researcher (the walking paths were not predefined for participants to allow capturing different environmental features), or (b) participants’ homes or their neighbourhood (for OA2, OA3, OA6 and P8) for 1–2 hours with no researcher in attendance. Data collection in outdoor environments was performed during daylight hours. Two participants walked with a cane/stick at all times and during walking outside home. OA3 and OA6 were living in the same home as marked by $\dagger$ in tables.

### Preprocessing

Gait/ambulatory bout definition is highly inconsistent in the literature, but is often defined as any walking episode $\ge$3 steps [6]. In the MAGFRA-W dataset, FPV data attributed to level walking bouts $\ge$3 steps (stairs ascending/descending episodes were excluded) were taken into account for annotation. FPV data collected during short pauses/stances between longer walks were not necessarily excluded. Frames attributed to the identified gait bouts were sampled at 6 Hz using MATLAB R2019b. Compared to 1/15 Hz in previous work [24], this sampling rate was appropriate to capture changes in environment during gait. FPV data for OA2 and OA3 (marked by $\star$ in tables) were accidentally collected with a lower sampling rate (resulting in a smaller quantity of annotated images, Table 1) and a higher resolution. Therefore, the subsequent frames were resized to align with the rest of data. Additionally, OA2, OA3 and OA6 wore the camera upside-down (marked by $\star$ in tables). Subsequently, a rotation was applied to permit comparisons with other data.

All sampled frames and image patches used for models development are in the RGB color space (e.g., 3$\times$1920$\times$1080), however, for simplicity, ’3$\times$’ is removed here when describing the dimensions.

### Considerations for the framework’s structure and annotation of MAGFRA-W data

#### Two-layer framework vs end-to-end approach

**Fig. 2:**
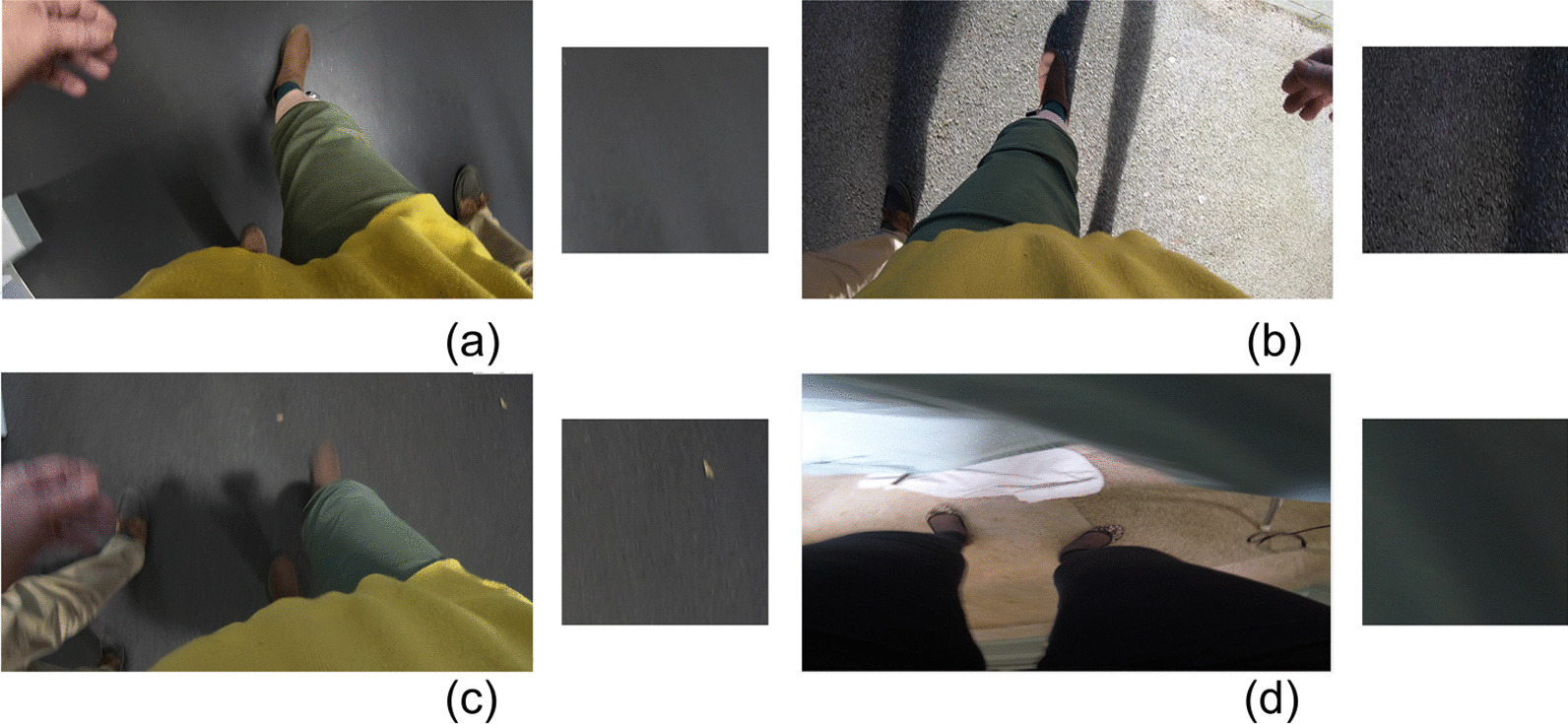
Patches cropped from right or left parts of sample frames: **a** laminate flooring (high-friction material), **b** asphalt, **c** carpet (high-friction material), **d** partial view of furniture. Although the type of the walking surfaces are different, the $453\times 453$ patches are very similar in terms of color and texture. *EgoPlaceNet* was adopted to classify frames into outdoor and indoor before terrain type identification to improve the framework’s performance

**Fig. 3:**
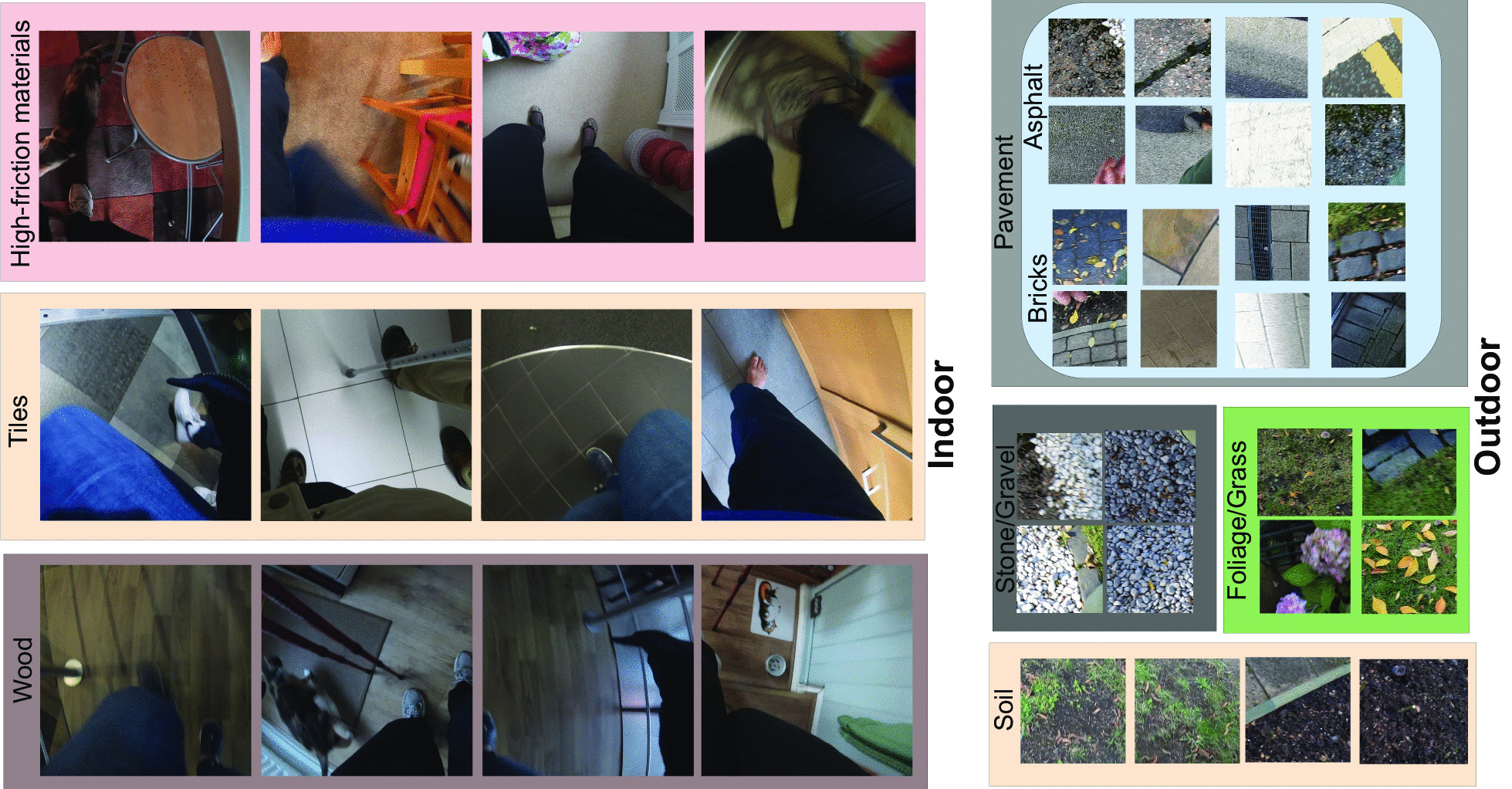
Sample patches from MAGFRA-W dataset. Outdoor patches were cropped at (267,0) and (1200,0) from the $1920\times 1080$ outdoor frames during gait. $1080\times 1080$ regions were cropped from upper left and right corners for indoor scenes. These dimensions were carefully selected to be compatible with the datasets used to train *EgoTerrainNet*-Outdoor and -Indoor

**Fig. 4:**
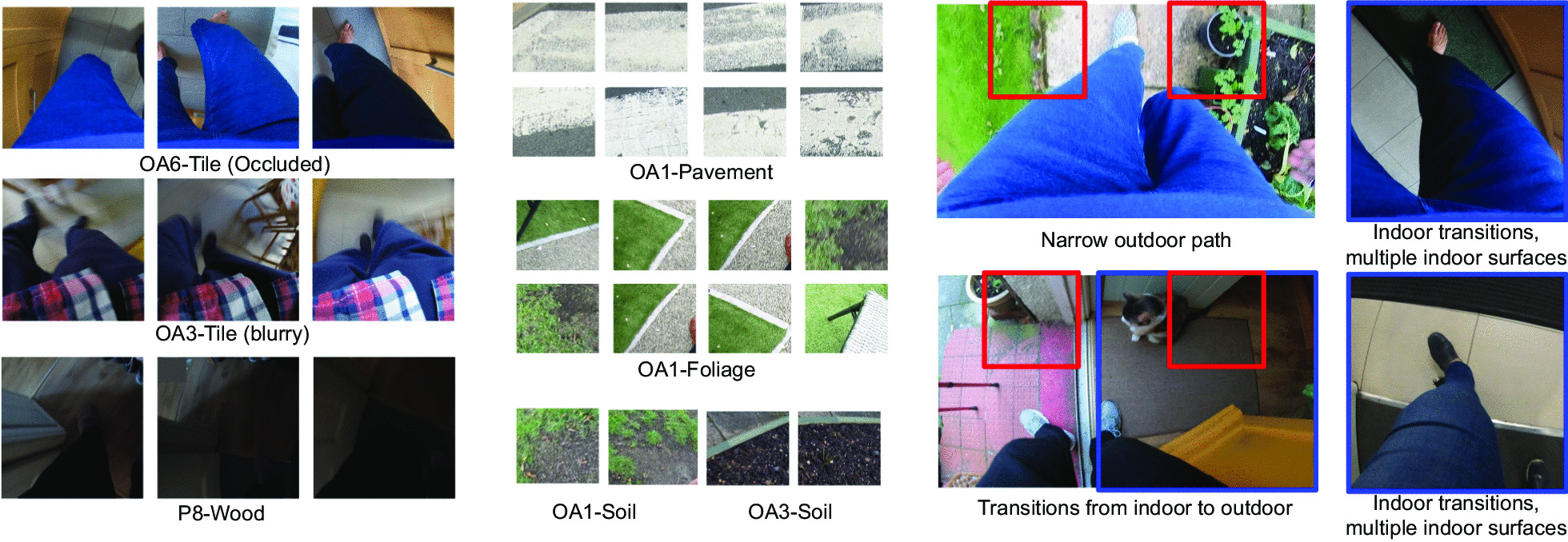
Sample frames/patches illustrating conditions challenging the performance of the proposed framework

Depending on the phase of gait (e.g., left heel strike in Fig. 1) and camera angle with respect to the frontal plane, a portion of the frames captured by a waist-mounted camera can be obscured by lower extremities and/or hands (see Figs. 2, 3, and 4). For instance, in Fig. 4-narrow *outdoor* path, a considerable portion of the frame is covered by participants’ blue jeans. In this case, it can be hypothesized that the color of pants/clothing may impact the prediction of an end-to-end model when the full frame, rather than its specific regions, is fed as input.

To address this, frames were investigated to identify robust regions in terms of the provision of terrain-related visual features. Two 453$\times$453 patches cropped at (267, 0) and (1200, 0) in 1920$\times$1080 frames (Fig. 1) were initially considered as representatives of surfaces underneath the participants’ left and right feet, respectively. These two patches were primarily cropped from all frames attributed to gait bouts. Considering the belt-mounted cameras’ field of view, the cropping parameters (e.g., the upper left corner coordinates, dimensions) were selected empirically to obtain regions with low overlap with upper/lower extremities during walking, but high overlap with walking surfaces.

From visual inspection of cropped patches, it was observed that 453$\times$453 regions attributed to different indoor and outdoor surfaces can resemble each other closely in terms of colour and texture (Fig. 2), which may lead to a low classification accuracy. Moreover, it was noticed that due to the higher complexity of indoor scenes (compared to outdoor scenes), there could be a higher likelihood of overlap between the two 453$\times$453 indoor patches with objects occluding views of the terrain such as walls and cabinets (Fig.  3). Thus, two larger 1080$\times$1080 regions cropped at (0, 0) and (840, 0) (Fig. 1 and Fig. 3) were considered as better representatives of indoor terrains. The smaller outdoor and larger indoor patches were also more similar to the images in GTOS and Material in Context (MINC) [36]) datasets, respectively, which were further considered to form an independent training dataset (discussed in "Assessing and augmenting models’ generalizability").

The aforementioned points necessitated the development of a two-layer framework, rather than an end-to-end approach (8-class classification considereing all terrain types), to first categorize frames based on their location into indoor and outdoor classes. The first-layer’s (i.e., *EgoPlaceNet* model) prediction further determines the frames’ regions that need to be cropped and fed into the second layer (i.e., *EgoTerrainNet*-Outdoor or -Indoor models) for terrain type identification (see Fig. 1).

#### Annotation of MAGFRA-W FPV data

Sampled frames (see subsection "Preprocessing") were further considered for the preparation of the ground truth data. To maintain 1:1 aspect ratio for each frame, a 1080$\times$1080 region was randomly cropped either from the top right or top left corners of the down sampled 1920$\times$1080 frames (1 crop for each frame, e.g., the blue square in Fig. 1). These crops were further annotated as outdoor (n=10,875) and indoor (n=6,099) depending on the enclosed scene to form the ground truth data for *EgoPlaceNet* (Table 1). Moreover, taking into account the criteria stated in "Considerations for the framework’s structure and annotation of MAGFRA-W data", 453$\times$453 and 1080$\times$1080 regions were cropped from outdoor (at (267,0) and (1200,0)) or indoor frames (at (0,0), (840,0)), respectively (e.g., red squares for outdoor scenes as shown in Fig. 1). To form the ground truth data for *EgoTerrainNet*s, outdoor crops were further annotated as: (a) pavement (e.g., outdoor tiles, bricks, asphalt and cement), (b) gravel/stone (including pebble, shale), (c) soil, (d) grass/foliage; and the indoor patches were grouped as (a) high friction materials (including carpet, fabric, laminate flooring, gym surfaces), (b) indoor tiles, and (c) wood (Fig. 3 and Table 1). Less than two patches were annotated for frames that either considerably overlapped with non-terrain materials (e.g., walls), had *fully* occluded field of view, or with unknown terrain type due to poor lighting ($\approx 7\%$ and $11\%$ of outdoor and indoor patches remained unlabeled, respectively). All image patches were extracted automatically using MATLAB R2019b.

#### Assessing and augmenting models’ generalizability

As discussed in "Background" and considering the results in Additional file 1: Materials (I), high accuracies obtained from holdout and k-fold approaches may not necessarily indicate model’s generalizability and robustness against environmental and/or inter-individual differences. Moreover, while MAGFRA-W data possesses high intra-class variance (see Fig. 3), surfaces may differ significantly from one OA’s home to another (e.g., carpet comes in a wide range of colours, patterns, and textures). As collecting a sufficiently large dataset to capture this heterogeneity across OAs’ everyday environments may not be feasible, similar to our previous research work [21], we hypothesized that curating a training dataset from other (reasonably similar) databases could increase intra-class variance, in terms of textures, colours, geometry, lighting conditions and clutter, and reduce the possible propensity to sample size bias. Subsequently, the framework’s generalizability to unseen datasets could be improved. The procedure for curating the training dataset is discussed in "Assessing and augmenting models’ generalizability".

In the case of observing unsatisfactory results from the former approach, the LOSO cross-validation approach (similar to leave-one-dataset-out in [21]) was considered as the next best in the present study to evaluate the framework’s generalizability and robustness against inter-participant variance in the MAGFRA-W dataset (although higher accuracies compared to the first approach is expected to be achieved [15, 20, 21]).

The validation accuracies during training (holdout: 70$\%$ training, 30$\%$ validation, see "Experiments") were separately reported for each network in the framework.

*Independent training dataset* A separate dataset was curated from other resources including public datasets: MINC-2500 (or MINC here) [36], GTOS(-mobile) datasets [27, 32] (or ’GTOS’ here), and HUJI-EgoSeg (or ’EgoSeg’ here) [37, 38]. These datasets complement each other to address identification of various terrain types observed under free-living conditions^1^. For instance, while the MINC-2500 dataset does not contain images of asphalt, there are asphalt and stone asphalt classes in GTOS (which includes outdoor terrain patches only). Moreover, although there are 2,500 images of carpet, wood and tiles in MINC-2500, only a small proportion resemble the images that could be taken from a top-down view. Considering a large proportion of images in MINC-2500 are irrelevant to MAGFRA-W (e.g. furniture, or cabinet in class ’wood’), only relevant images from MINC-2500 were selected (e.g., 445/2500 from wood as hardwood flooring, Additional file 2: Materials (II)).

As mentioned in  "Background", images in GTOS were collected while the camera-ground distance is much smaller than the height of the waist-mounted camera. This field of view resulted in very low complexity and intra-class variance in GTOS (e.g., pedestrian’s feet were not observed in the image) compared to the higher view in MAGFRA-W and may reduce the prospects for generalizability to everyday terrains. Although there are 40 different classes of outdoor terrains in GTOS, differentiating between each may not provide relevant additional information for gait assessment and free-living FRA. For example, separate GTOS classes of asphalt, cement, or pavement bricks may not result in substantially different walking patterns. Thus for the purpose of this study, images from the relevant classes were combined.

To further address the limitations of MINC and GTOS, the suitability of several FPV-based datasets (e.g. EPIC-Kitchens 2018 [39]) was examined. Among public FPV datasets, HUJI-EgoSeg was considered a suitable candidate, as the camera wearers walked in diverse outdoor environments. HUJI EgoSeg video data were collected from a head-mounted GoPro Hero3+ camera during a range of activities (e.g., walking, riding bus, driving). After resizing 720p frames to 1920$\times$1080, patches of 453$\times$453 were cropped from the lower-central, right, and left parts of the resized frames. Considering head-mounted cameras may not provide a consistent view of terrain, only a handful were annotated and included.

As GTOS and MINC datasets contain no images of snow/slush-covered terrains, a smartphone at waist level was used to capture videos of slush- or snow-covered terrains by the authors. Patches (453$\times$453) were cropped from the right and left corners of the frames and added to the training dataset. Although this snow/slush class may not have representatives in the test dataset (MAGFRA-W), snow-covered terrains are frequently observed in regions with low average yearly temperature, impact gait patterns and are a potential risk factor for falls. Therefore, adding this class would improve the framework’s relevance and generalizability.

Overall, 3,651 and 5,773 image patches were extracted from the aforementioned datasets to form training datasets for indoor and outdoor surfaces, respectively. The distribution of patches extracted from different datasets as well as sample patches for snow/slush has been shown in Additional file 2: Materials (II). The open access image/FPV datasets discussed here can be accessed and viewed from their corresponding data repositories.

### Pre-trained ConvNets

Considering the size of the curated training dataset and MAGFRA-W (also used for training in the LOSO approach), training a deep ConvNet from scratch was not feasible. Therefore, the transfer learning approach [40] was considered. This subsection discusses the criteria for selecting the backbone models (ConvNet pre-trained on a large-scale dataset) for *EgoPlaceNet* and *EgoTerrainNet*.

For applications in prosthestics and exoskeletons, the real-time detection of environmental features is a critical part of the control loop. While on-device detection of environmental features is not necessary for the purpose of FRA, this allows processing of frames without the need for storing videos, and may subsequently mitigate privacy and ethical issues associated with FPV data use. By benchmark analysis of state-of-the-art deep neural network architectures (in terms of accuracy, size of the learnable parameters, memory usage, computational complexity using the floating-point operations, and inference time), SqueezeNets, MobileNets, ResNet-18, GoogLeNet, and AlexNet achieved optimal real-time performance, while no significant relationship between model complexity and recognition accuracy was reported [41]. Building upon the idea of depth-wise separable convolution from MobileNetV1 [42], MobileNetV2 pushed the state of the art for mobile image classification [43] using the inverted residual with linear bottleneck as a novel layer module. This resulted in faster and more accurate performance while using $\approx 30\%$ fewer parameters compared to MobileNetV1. Therefore, MobileNetV2 pretrained on ImageNet [44] was considered as the initial candidate for backbone models in our study.

*Considerations for EgoPlaceNet*: In contrast to ImageNet categories, where indoor and outdoor scenes were not separated, images in Places365 dataset [45] were categorized into indoor and outdoor macro-classes (e.g., indoor and outdoor categories for ice skating rink) and the models were trained on millions of scene photographs. Therefore, deep networks trained on this dataset have learned different feature representations for a wide range of indoor and outdoor images compared to ImageNet, and hypothesized to be a better candidate for *EgoPlaceNet* (where the desired task is similar to classifying scenes) resulting in higher accuracies. Among the available pre-trained deep models on Places365 dataset^2^, ^3^, AlexNet (with over 60 million parameters for $227\times 227$ images, 8 layers, [46]) and GoogLeNet ($\approx$12 times fewer parameters compared to AlexNet, 22 layers [47]) models were considered as the backbone model for *EgoPlaceNet*.

Fine-tuning procedures for all models are discussed in "Experiments".

### Experiments

For *EgoTerrainNet*-Outdoor and -Indoor versions, MobileNetV2’s were fine-tuned using the curated training dataset discussed in "Assessing and augmenting models’ generalizability", by replacing the last fully connected layer and the final classification layer of the network.

The GoogLeNet pre-trained on Places365, was first fine-tuned on indoor and outdoor images in the curated training dataset (described in "Assessing and augmenting models’ generalizability") . The subsequent model is referred to as *EgoPlaceNet*.v1 in the present study. Considering the evaluation criteria detailed in "Assessing and augmenting models’ generalizability", after observing the *EgoPlaceNet*.v1’s results when tested on the MAGFRA-W datset, LOSO cross-validation (*EgoPlaceNet.LOSO*$_n$, $n=\{1,\dots ,9\}$) was further performed to investigate models’ robustness against inter-participant variations. To implement this, the GoogLeNet-Places365 was fine-tuned based on the dataset acquired from 8 participants, and tested on the remaining data from one participant.

The GoogLeNet-Places365 models were fine-tuned by freezing the weights of 10 earlier layers in the network according to preliminary results. The improved results obtained by unfreezing the weights of more layers than solely the last fully connected layer (considered for *EgoTerrainNet*s fine-tuning) was likely due to the fewer number of classes in the binary classification approach (vs 5 for *EgoTerrainNet*-Outdoor), and thus, the availability of more samples in each class.

Depending on the model (i.e., $EgoPlaceNet.LOSO_n$, *EgoPlaceNet.v1*, *EgoTerrainNet*-Outdoor, *EgoTerrainNet*-Indoor) the relevant training dataset (e.g., indoor or outdoor, as discussed in "Assessing and augmenting models’ generalizability", or remaining 8 OAs for $EgoPlaceNet.LOSO_n$) was randomly divided into training (70$\%$) and validation (30$\%$) with images resized to 224$\times$224. Experiments were performed on a workstation (Intel(R) Core (TM)i7-6700, 3.4GHz with Nvidia GeForce GTX 750 Ti), with MATLAB R2019b. The mini-batch sizes of *K* = 10 and *K* = 64 were used (due to the limited memory), for *EgoPlaceNet* and *EgoTerrainNet*, respectively. The training procedure was terminated manually when the model performance stopped improving (by monitoring the loss/accuracy in the performance plot) to avoid overfitting. Validation patience was set to 20.

The initial learning rate of $\gamma = {0.01}$ for *EgoTerrainNet*-Outdoor and -Indoor and $\gamma = 3e$-4 for *EgoPlaceNet* models resulted in the best validation accuracies. Stochastic gradient descent with momentum was considered as the optimization method. Moreover, the following hyperparameters were employed: momentum: 0.9, L2 Regularization: 1*e*-4, gradient threshold method: L2 norm, and decay rate of 0.0005. To further address the problem of a small dataset, improve the generalization of the network, and prevent the models from overfitting and memorizing the details of the training images, data were augmented by including random crops, translation, rotation $\in [-20 +20]$ deg (accounting for changes in camera orientation during gait) and vertical reflection/flip over y axis. Considering the viewpoints of images in the training dataset as well as data captured by a belt-mounted camera, horizontal reflection was not considered for augmentation. Using MATLAB data augmentation object and the aforementioned transformations, one randomly augmented version of each image was used during each epoch of training. No further manual data augmentation was preformed.

## Results

At the end of the training process, the validation accuracies of 93.97$\%$, 98.19$\%$ (mean-over-participants), 99.23$\%$, and 85.26$\%$ were achieved for *EgoPlaceNet*.v1, *EgoPlaceNet.LOSO*$_n$, *EgoTerrainNet*-Outdoor and *EgoTerrainNet*-Indoor, respectively.

**Table 2:**
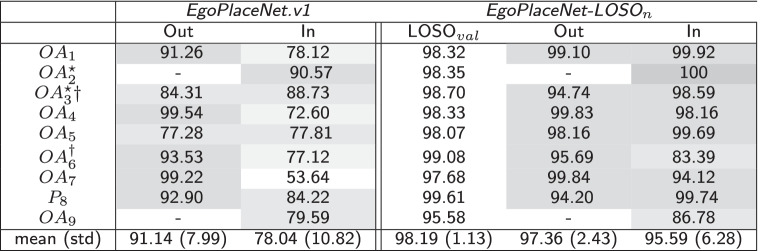
Results for 1. *EgoPlaceNet.v1* (fine-tuned on the selected training dataset from MINC+HUJI EgoSeg+GTOS) when applied to MAGFRA-W (validation accuracy at the end of the training process: 93.97$\%$), and 2. *EgoPlaceNet-LOSO*$_n$ for participant *n*. LOSO$_{val}$ indicates the validation accuracy at the end of the training process for each model. Darker shades of grey indicate higher per-class accuracies.

The *EgoPlaceNet*.v1 resulted in test accuracies of 91.14$\%$ (std: 7.99$\%$) and 78.04$\%$ (std: 10.82$\%$) (Table 2) for the detection of outdoor and indoor scenes in MAGFRA-W, respectively. However, these rose to 97.36$\%$ (std: 2.43$\%$) and 95.59$\%$ (std: 6.28$\%$) when LOSO cross-validation was performed (9 models, *EgoPlaceNet.LOSO*$_n$).

**Table 3:**
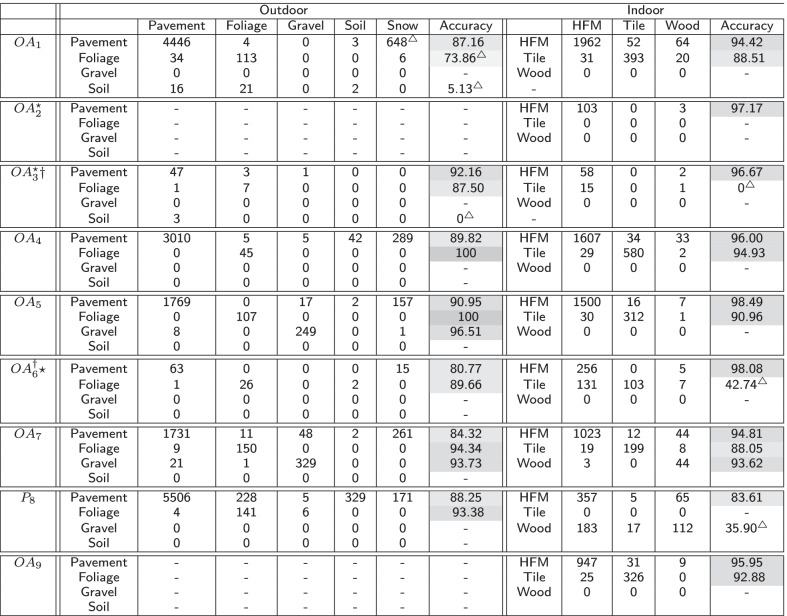
Confusion Matrices at participant level: for *EgoTerrainNet*-Outdoor and -Indoor, MobileNetV2’s pre-trained on ImageNet dataset were fine-tuned. The validation accuracies (during training) for -Outdoor and -Indoor versions were 99.23 and 85.26, respectively. $\star :$ camera was unintentionally mounted upside-down by the participants or was set to take photos (not videos), $\dagger :$ Participants living in the same home, HFM: high-friction materials, $\bigtriangleup$: cases that are discussed in "Deeper analysis of lower accuracies ".Darker shades of gray indicate higher per-class accuracies

Confusion matrices and per-class detection accuracies for each participant were examined separately to better assess the impact of inter-participant differences, colour of clothing, camera placement, and environmental features on models’ performance (Table 3). Therefore, although the distribution of image patches over classes were different for participants, due to the aforementioned points, the mean-over-participant detection accuracy for each class was considered as a more suitable metric compared to overall detection accuracy for each class (e.g., total true positives from all participants for class ’pavement’/total number of patches labeled as ’pavement’ from all participants). In addition to mean-over-participant accuracies, standard deviation (std) measures were reported for scene/terrain classes for which data from $\ge$3 individuals were available.

*EgoTerrainNet*-Outdoor exhibited satisfactory performance (mean-over-participant test accuracies)for the identification of pavement (87.63$\%$ (std: 3.97)), grass/foliage (91.24$\%$ (std: 9.00)), and stone/gravel (95.12$\%$ (std: 1.96)). However, it failed to detect soil with high acuracies for OA1 and OA3 (Table 3). *EgoTerrainNet*-Indoor detected high-friction materials including carpet and laminate flooring with a high accuracy (mean over participants: 95.02$\%$ (std: 4.48)). However, the mean-over-participant accuracies drastically decreased to 71.15$\%$ (std: 36.25) and 64.76$\%$ for tiles and wood, respectively. While tile identification accuracy was high in most participants (OA1: 88.51$\%$, OA4: 94.93$\%$, OA5: 90.96$\%$, OA7: 88.05$\%$, OA9: 92.88$\%$), the results of OA3 (0$\%$) and OA6 (42.74$\%$) decreased the mean accuracy for tile detection. It was interesting as the color of tiles in OA3’ and OA6’s home was ’grey’, and similar to some sample patches from other participants’ data (see Figs. 3 and 4). Similarly, wood identification achieved a high and low accuracies for OA7 (93.62$\%$) and P8 (35.90$\%$), respectively.

### Deeper analysis of lower accuracies

Results marked by $\bigtriangleup$ in Table 3 are further discussed in this subsection.

First, pavement is mainly confused with soil (e.g., in P8) and snow (e.g., OA1). In OA1’s data, the 453$\times$453 patches overlapped with regions of pavement with white paintings/street signs (Fig. 4), which could be confused with snow. Moreover, many images in asphalt and soil classes in the curated training dataset for *EgoTerrainNet*-Outdoor ("Assessing and augmenting models' generalizability" Section) share similar visual features such as colour and texture, which partially explains the aforementioned confusion.

Samples in soil were mainly confused with grass/foliage in OA1. In OA1’s data, soil was frequently mixed with or covered by grass/foliage (Fig. 4). Considering the lack of a standard definition for the annotation of patches, either grass/foliage or soil may have been assigned to those patches, which may explain the subsequent results.

The camera’s field of view during free-living data collection in OA3’s and OA6’s home was heavily occluded by participants’ clothing (e.g., blue pants/jeans). In addition to covering the tiles texture, the participants’ clothing likely confused *EgoTerrainNet*-Indoor to classify tiles as high-friction material (e.g., fabric/carpet) (see Fig. 4). The images were also blurry in many cases (Fig. 4), concealing the texture of tiles in the subsequent images.

The lower wood detection accuracy for P8, compared to OA7, could be attributed to poor lighting conditions in the former’s home (Fig. 4). The image patches attributed to ’wood’ for this participant were mainly categorized as high-friction materials, likely because the texture of wood was not differentiable in the subsequent 1080$\times$1080 patches due to poor lighting condition.

## Discussion

This paper proposes an egocentric vision-based framework to automatically detect indoor and outdoor level walking surfaces. To the best of the authors’ knowledge, this work is the first to present a deep learning-based model tested on OAs’ everyday FPV data towards the development of a context-aware free-living FRA.

The MAGFRA-W dataset offers a considerable diversity in terms of terrain types and visual characteristics (presence of pets and walking aids, clothing in different colours), lighting conditions, feet appearance (e.g., shoes with different colours, barefoot, socks, slippers) leading to more ecologically valid classification results compared with data collected in controlled conditions. After investigating the participants’ FPV data and other relevant public datasets, a two-layer structure was considered superior to an end-to-end approach for terrain type identification. Subsequently, the training and test datasets were prepared according to this hypothesis. Overall, it can be concluded that aggregating *EgoPlaceNet* trained on outdoor-indoor images captured by a belt-mounted camera, followed by *EgoTerrainNet*s trained on an independent dataset leads to the best terrain identification performance in terms of accuracy and generalizability.

To train and test *EgoPlaceNet* and *EgoTerrainNet*s, several approaches could have been considered: (a) holdout and k-fold cross-validation, (b) LOSO using MAGFRA-W, or (c) using MAGFRA-W as the test dataset and incorporating an independent (but sufficiently similar) training dataset for fine-tuning deep models. Considering that the discrepancy between the distributions of training and test datasets in approach (c) avoids the generation of unrealistically high accuracies, we considered this option to be superior. Furthermore, option (c) is aligned with our previous research work [21] and represents a pragmatic picture of the proposed framework’s generalizability. Option (b) was considered as the next best to evaluate the framework’s robustness against inter-participant differences.

To form an independent training set for approach (c), relevant images (or frames) from different datasets (e.g., MINC-2500, HUJI EgoSeg, and GTOS) were selected. The subsequent fine-tuned ConvNets on this dataset, i.e., *EgoTerrainNet*-Outdoor and -Indoor, were applied to specific regions of outdoor ($453\times 453$) and indoor ($1080\times 1080$) frames. Promising results exhibited the models’ generalizability to detecting a broad range of terrains. Although the sample size for the curated dataset was relatively small (9,424 images overall), the results indicate that this dataset captured a high variations of texture, colour, and shape in everyday scenes, which bypasses the requirement for prolonged data collection from a large cohort of OAs to form a heterogeneous training dataset. This approach also outperformed the models that were solely fine-tuned on one dataset (e.g., GTOS or MINC, as shown in Additional file 3: Materials (III)).

*EgoPlaceNet*.v1 achieved 91.14$\%$ and 78.04$\%$ detection accuracies for outdoor and indoor scenes, respectively. The relatively poor performance of this binary classifier supports the hypothesis that an end-to-end approach, i.e., an 8-class classification problem (more complex compared to the binary classification) may not exhibit a robust performance if option (c) is considered. On the other hand, high *EgoPlaceNet.LOSO*$_n$ accuracies ($\ge$95$\%$ for both indoor and outdoor scenes, Table 2) confirm the models’ robustness against variations in participants’ characteristics, camera view and partial occlusions (e.g., lower extremities, walking aids).

High detection accuracies were consistently observed for pavement, gravel, grass/foliage, and high-friction materials for all participants. Among the outdoor terrain types, soil had the lowest detection accuracy as well as a low per-class quantity in MAGFRA-W (only 42 samples, see Table 1). Additionally, no sample of snow was found in the MAGFRA-W dataset. These points necessitate further investigation of *EgoTerrainNet*-Outdoor’s performance using a more inclusive test dataset in future studies. Moreover, while tiles (in different patterns and colors such as grey, white, see Fig. 3) in public environments were detected with high accuracies (OA1, OA4-5, OA7, OA9; ranging from $\approx 88\%$ to $\approx 95\%$), in-home tiles (mostly grey) captured in OA3’s and OA6’s home were mainly confused with high-friction materials. The same trend was observed for ’wood’, which was detected with $93.62\%$ and 35.90$\%$ accuracies for OA7 (public environment) and P8 (in-home), respectively. As detection of wood and indoor tiles require capturing fine details of terrain textures, partially-obscured views as well as blurry and/or dark images due to dim lighting conditions in in-home settings were considered as the primary reasons for this inferior performance. A similar phenomenon was observed in other studies [48, 49], where image blur/noise led to a considerable drop in classification accuracies. Methods have been proposed to exclude or skip blurry images [28, 29], at the expense of heavier computational demand. Other works have suggested that classification performance of deep architectures could be improved by fine-tuning the models on blurry images [49]. In [50], authors jointly trained a deblurrer combined with a high-level computer vision network. Therefore, the integration of similar pipelines into the proposed framework in the present study may augment the performance of *EgoTerrainNet*-Indoor.

The backbone deep models considered here (i.e., MobileNetV2 and GoogLeNet pretrained on ImageNet and Places365 datasets, respectively) were selected based on multiple criteria and previous comparison studies (discussed in subsection "Pre-trained ConvNets"), and exhibited satisfactory performance in terms of detection accuracy. Further investigation using larger-scale datasets is required to identify the optimal deep architecture addressing terrain type identification in the wild. Moreover, the employed parameters (e.g., learning rate, number of frozen layers for transfer learning) were selected based on some preliminary numerical analyses and may be tuned by performing deeper analyses in future studies.

While the collection of FPV data in controlled conditions facilitates the process of image annotation by providing high quality and consistent data, the complex nature of everyday scenes captured in the MAGFRA-W dataset challenged the process of image patch annotation. First, a subset of image patches ($7\%$ and $11\%$ of the extracted outdoor and indoor patches, respectively) remained unlabelled due to their significant overlap with non-terrain materials such as walls, dim lighting conditions, or obscured views. Therefore, although the accuracies for *EgoPlaceNet* and *EgoTerrainNet* were calculated separately, the overall framework’s accuracy (the sequential approach) could not be reported. The addition of class ’others’ [36] in the training dataset could have been considered to address this limitation, however, the preparation of relevant samples collected from the top-down view to form this class was out of the scope of the present study. Secondly, in addition to mixed surfaces (e.g., soil and grass in Fig. 4), transitions between different locations and surfaces (see Fig. 4) challenged the annotation of ground truth data. For example, while only one label was attributed to each $1080\times 1080$ patch, in Fig. 4 (right panel) each foot is placed on a different surface. Subsequently, both tiles and high-friction materials could be considered as valid labels for the patch. Such a discrepancy in the annotations could introduce errors to the reported results. This issue occurred less frequently during the annotation of outdoor patches, as due to their smaller size (compared to indoor patches) the enclosed outdoor terrain type was generally more consistent. Considering a belt-mounted camera’s field of view, a separate 453$\times$453 region was expected to represent the terrain type around each foot in outdoor scenes. However, there were exceptions. For instance in Fig. 4, the OA is walking on a narrow ’brick-covered’ ($\in$pavement) surface, and the right and left patches partially overlap with foliage, which is irrelevant to the walking surface type. By integrating the spatial and temporal (embedded in optical flow) information in our previous research work [21], the *FootChaser* framework was proposed to localize feet in the video data captured from a belt-mounted camera for the purpose of gait assessment. Therefore, rather than cropping frames’ fixed regions (considered in the present study), the integration of *FootChaser* model into the proposed framework is expected to allow cropping more specific regions (with varying sizes) of frames in the proximity of each localized foot. This may permit a more accurate identification of walking surfaces.

Considering the preliminary results of Weiss et al. [12] and evidence reported in Additional file 1: Materials (I) regarding the feasibility of stair walking detection using IMU data alone, the present study focused on vision-based detection of level walking surfaces. While comparing the performance of IMU- and FPV-based models for the task of level walking surface identification requires a rigorous assessment, the inferior performance of the incorporated IMU-based approach^4^ for the detection of gravel, grass and paved/flat surfaces (<50% LOSO accuracies, Additional file 1: Materials (I)), along with *EgoTerrainNet*-Outdoor’s satisfactory detection accuracies for the same outdoor classes (>87%, see "Results" Section), imply that egocentric vision-based models generally lead to more promising results for the aforementioned task, and thus, can be integrated to improve the interpretability of commonly used IMU-based FLDBs. However, incorporating an additional sensor modality (i.e., camera) along with IMU(s) may negatively impact compliance in larger-scale field studies (e.g., due to ethical/privacy concerns, obtrusive sensor placement). While further testing to assess the acceptability of the proposed vision-based framework by older populations is beyond the scope of the present study, subsequent efforts may focus on mitigating potential ethical/privacy issues associated with egocentric vision data use. This may be achieved using light-weight models (including those employed in the present paper) to enable automated processing without the need to store videos. Overall, the fast-paced advancements in miniaturized wearable sensor technologies combined with deep learning models with low computational demand, are promising for advancements in egocentric vision methods in the area of neurorehabilitation engineering (e.g., [24, 51, 52]). Smaller cameras with high on-board processing power, expected in the near future, can facilitate unobtrusive sensor modalities while preserving older adults’ privacy through on-device processing at the same time.

Considering IMU data were collected along with FPV data in the MAGFRA-W dataset, the impact of environmental features on a comprehensive list of IMU-based gait-related FLDBs (e.g., spatiotemporal gait) [6] will be investigated in both faller and non-faller groups in our future studies. Moreover, similar to multimodal deep models proposed to address activity recognition [53, 54], temporal and spatial information during gait can be examined simultaneously by a hybrid multi-stream network trained on both FPV (including still images and optical flow) and IMU data. By examining potential interactions between intrinsic and extrinsic factors captured in different modality types, the subsequent framework may outperform the models trained solely on FPV data for the task of terrain type identification. While demographic factors (e.g., gender, history of falls, age) have minimal impact on the spatial data (still frames) in the MAGFRA-W dataset, gait-related temporal data can be impacted by such factors. Therefore, addressing the aforementioned multimodal approaches requires a balanced dataset in terms of the demographic characteristics, which will be achieved in the future phases of the project.

In our future work, other details of the walking surfaces will be considered. For instance in OA4’s multimodal data (IMU and FPV), 1 naturally-occurring (hit and bump) misstep was automatically detected by applying an IMU-based model, where a light pole was visually verified as the environmental fall risk [15]. Therefore, algorithms to detect such a static obstacle, as well as other tripping hazards (including dynamic obstacles such as pedestrians and pets, Fig. 3) and cracks in pavement [55] will be considered to provide complementary information on the properties of environment, towards a comprehensive context-aware free-living gait and fall risk assessment method. The automated identification of contexts associated with falls (and missteps [15]) using egocentric vision data would increase the interpretability of IMU-based FLDBs and address more specific intervention strategies, including the environmental modification (e.g., removing obstacles, securing fall areas, using non-slip flooring materials) as well as rehabilitation interventions (e.g., training to negotiate obstacles), which can potentially reduce the frequency of future falls in older adults. For instance, if a high frequency of slips (a form of misstep) is observed while walking on indoor tiles, non-slip flooring materials can be integrated to avoid future imbalance events.

## Conclusions

Overall, encouraging results suggest that the integration of wearable cameras as well as deep learning approaches can provide objective information on the properties of walking surfaces, towards context-aware FLDBs for gait and fall risk assessment in the wild. Considering IMU data were collected along with FPV data in MAGFRA-W, the impact of environmental features on IMU-based gait-related FLDBs will be investigated in our future works.

## Supplementary Information

**Additional file 1.** Preliminary results for IMU-based surface type identification.

**Additional file 2.** The independent training dataset curated from multiple sources.

**Additional file 3.** Preliminary terrain type identification results using MINC-2500 and GTOS datasets.

## Data Availability

The anonymized pre-processed data from MAGFRA-W dataset may be made available to interested researchers upon reasonable request. MINC-2500, HUJI-EgoSeg, and GTOS are all public datasets that can be accessed through their corresponding data repositories.

## Abbreviations

FLDB: Free-living digital biomarkers
IMU: Ineretial measurment unit
MAGFRA: Multimodal Gait and Fall Risk Assessment
OA: Older adult
FRA: Fall risk assessment
LOSO: Leave-one-subject-out
FPV: First person vision
GTOS: Ground Terrain in Outdoor Scenes dataset
MAGFRA-W: Multimodal Gait and Fall Risk Assessment in the Wild
MAGFRA-C: Multimodal Gait and Fall Risk Assessment in Clinic
MINC: Material in Context datseset

## Footnotes

1 Additional file 3:Materials (III) shows the preliminary test results of *EgoTerrainNet*-Outdoor and -Indoor fine-tuned on (a) a subset of GTOS and (b) all 2,500 images of ’wood’, ’carpet’, and ’tiles’ in MINC-2500, when applied to MAGFRA-W. The results suggest that one dataset alone may not result in generalizable models.

2 https://github.com/CSAILVision/places365

3 https://www.mathworks.com/help/deeplearning/ref/googlenet.html

4 The incorporated IMU-based approach exhibited satisfactory performance in our previous research [15] addressing a different research question, and was hypothesized to be a suitable baseline candidate for the purpose of terrain type identification. This approach demonstrated satisfactory performance for the identification of stair walking episodes, as discussed in Additional file 1: Materials (I).

## References

[1] World Health Organization, *WHO global report on falls prevention in older age*. World Health Organization, 2008.

[2] Berg, R.L. and Cassells, J.S., 1992. Falls in older persons: risk factors and prevention. In *The second fifty years: Promoting health and preventing disability*. National Academies Press (US). 25144081

[3] Rubenstein LZ (2006). Falls in older people: epidemiology, risk factors and strategies for prevention. Age Ageing.

[4] Shumway-Cook A, Brauer S, Woollacott M (2000). Predicting the probability for falls in community-dwelling older adults using the timed up & go test. Phys Ther.

[5] Schoene D, Wu SM-S, Mikolaizak AS, Menant JC, Smith ST, Delbaere K, Lord SR (2013). Discriminative ability and predictive validity of the timed up and go test in identifying older people who fall: systematic review and meta-analysis. J Am Geriatr Soc.

[6] Nouredanesh M, Godfrey A, Howcroft J, Lemaire ED, Tung J (2020). Fall risk assessment in the wild: a critical examination of wearable sensors use in free-living conditions. Gait Posture..

[7] Del D, Galna B, Godfrey A, Bekkers EM, Pelosin E, Nieuwhof F, Mirelman A, Hausdorff JM, Rochester L (2017). Analysis of free-living gait in older adults with and without Parkinson’s disease and with and without a history of falls: identifying generic and disease specific characteristics. J Gerontol A Biol Sci Med Sci.

[8] Iluz T, Gazit E, Herman T, Sprecher E, Brozgol M, Giladi N, Mirelman A, Hausdorff JM (2014). Automated detection of missteps during community ambulation in patients with Parkinson’s disease: a new approach for quantifying fall risk in the community setting. J Neuroeng Rehabil.

[9] Mancini M, Schlueter H, El-Gohary M, Mattek N, Duncan C, Kaye J, Horak FB (2016). Continuous monitoring of turning mobility and its association to falls and cognitive function: a pilot study. J Gerontol A Biol Sci Med Sci.

[10] Twardzik E, Duchowny K, Gallagher A, Alexander N, Strasburg D, Colabianchi N, Clarke P (2019). What features of the built environment matter most for mobility? Using wearable sensors to capture real-time outdoor environment demand on gait performance. Gait Posture.

[11] Del Din S, Godfrey A, Galna B, Lord S, Rochester L (2016). Free-living gait characteristics in ageing and Parkinson’s disease: impact of environment and ambulatory bout length. J Neuroeng Rehabil.

[12] Weiss A, Brozgol M, Giladi N, Hausdorff JM (2016). Can a single lower trunk body-fixed sensor differentiate between level-walking and stair descent and ascent in older adults? preliminary findings. Med Eng Phys.

[13] Weiss A, Brozgol M, Dorfman M, Herman T, Shema S, Giladi N, Hausdorff JM (2013). Does the evaluation of gait quality during daily life provide insight into fall risk? A novel approach using 3-day accelerometer recordings. Neurorehabil Neural Repair.

[14] Ihlen EA, Weiss A, Bourke A, Helbostad JL, Hausdorff JM (2016). The complexity of daily life walking in older adult community-dwelling fallers and non-fallers. J Biomech.

[15] Nouredanesh M, Ojeda L, Alexander NB, Godfrey A, Schwenk M, Melek W (2022). Automated Detection of Older Adults’ Naturally-Occurring Compensatory Balance Reactions: Translation From Laboratory to Free-Living Conditions. IEEE J Translat Eng Health Medicine.

[16] Handelzalts S, Alexander NB, Mastruserio N, Nyquist LV, Strasburg DM, Ojeda LV (2020). Detection of real-world trips in at-fall risk community dwelling older adults using wearable sensors. Front Med.

[17] Hashmi MZUH, Riaz Q, Hussain M, Shahzad M (2019). What lies beneath one’s feet? Terrain classification using inertial data of human walk. Appl Sci.

[18] Hu B, Dixon P, Jacobs J, Dennerlein J, Schiffman J (2018). Machine learning algorithms based on signals from a single wearable inertial sensor can detect surface-and age-related differences in walking. J Biomech.

[19] Hu B, Li S, Chen Y, Kavi R, Coppola S (2021). Applying deep neural networks and inertial measurement unit in recognizing irregular walking differences in the real world. Appl Ergon.

[20] Nouredanesh M, Gordt K, Schwenk M, Tung J (2019). Automated detection of multidirectional compensatory balance reactions: a step towards tracking naturally-occurring near-falls. IEEE Trans Neural Syst Rehabilitation Eng.

[21] Nouredanesh M, Li AW, Godfrey A, Hoey J, Tung J. Chasing feet in the wild: a proposed egocentric motion-aware gait assessment tool. In: European Conference on Computer Vision, Springer; pp. 176–192 2018.

[22] Luo Y, Coppola SM, Dixon PC, Li S, Dennerlein JT, Hu B (2020). A database of human gait performance on irregular and uneven surfaces collected by wearable sensors. Sci Data.

[23] Stone EE, Skubic M (2013). Unobtrusive, continuous, in-home gait measurement using the microsoft kinect. IEEE Trans Biomed Eng.

[24] Taylor K, Reginatto B, Patterson MR, Power D, Komaba Y, Maeda K, Inomata A, Caulfield B. Context focused older adult mobility and gait assessment. In: Engineering in Medicine and Biology Society (EMBC), 2015 37th Annual International Conference of the IEEE, IEEE; pp. 6943–6946, 2015. 10.1109/EMBC.2015.7319989 26737889

[25] Wang W, Zhang B, Wu K, Chepinskiy SA, Zhilenkov AA, Chernyi S, Krasnov AY (2022). A visual terrain classification method for mobile robots’ navigation based on convolutional neural network and support vector machine. Trans Inst Meas Control.

[26] Julius Fusic S, Hariharan K, Sitharthan R, Karthikeyan S (2021). Scene terrain classification for autonomous vehicle navigation based on semantic segmentation method. Trans Inst Meas Control.

[27] Xue J, Zhang H, Dana K, Nishino K. Differential angular imaging for material recognition. In: Proceedings of the IEEE Conference on Computer Vision and Pattern Recognition, pp. 764–773, 2017.

[28] Anantrasirichai N, Burn J, Bull D (2014). Terrain classification from body-mounted cameras during human locomotion. IEEE Trans Cybern.

[29] Diaz JP, da Silva RL, Zhong B. Huang HH, Lobaton E. Visual terrain identification and surface inclination estimation for improving human locomotion with a lower-limb prosthetic. In: 2018 40th Annual International Conference of the IEEE Engineering in Medicine and Biology Society (EMBC), IEEE; pp. 1817–1820, 2018. 10.1109/EMBC.2018.8512614 30440748

[30] Sharif Razavian A, Azizpour H, Sullivan J, Carlsson S. Cnn features off-the-shelf: an astounding baseline for recognition. In: Proceedings of the IEEE Conference on Computer Vision and Pattern Recognition Workshops, pp. 806–813, 2014.

[31] Okafor E, Pawara P, Karaaba F, Surinta O, Codreanu V, Schomaker L, Wiering M. Comparative study between deep learning and bag of visual words for wild-animal recognition. In: 2016 IEEE Symposium Series on Computational Intelligence (SSCI), IEEE; pp. 1–8, 2016.

[32] Xue J, Zhang H, Dana K. Deep texture manifold for ground terrain recognition. In: Proceedings of the IEEE Conference on Computer Vision and Pattern Recognition, pp. 558–567, 2018.

[33] Nouredanesh M, McCormick A, Kukreja SL, Tung J. Wearable vision detection of environmental fall risk using gabor barcodes. In: 2016 6th IEEE International Conference on Biomedical Robotics and Biomechatronics (BioRob), IEEE; pp. 956–956, 2016.

[34] Nouredanesh M, Godfrey A, Tung J (2019). First-person vision-based assessment of fall risks in the wild, towards fall prevention in older adults. JCVIS.

[35] Nouredanesh M, McCormick A, Kukreja SL, Tung J. Wearable vision detection of environmental fall risk using gabor barcodes. In: Biomedical Robotics and Biomechatronics (BioRob), 2016 6th IEEE International Conference On, IEEE; pp. 956–956, 2016.

[36] Bell S, Upchurch P, Snavely N, Bala K. Material recognition in the wild with the materials in context database. In: Proceedings of the IEEE Conference on Computer Vision and Pattern Recognition, pp. 3479–3487, 2015.

[37] Poleg Y. Arora C, Peleg S. "Temporal segmentation of egocentric videos." In: Proceedings of the IEEE Conference on Computer Vision and Pattern Recognition, pp. 2537-2544, 2014.

[38] Poleg Y, Ephrat A, Peleg S, Arora C. Compact cnn for indexing egocentric videos. In: 2016 IEEE Winter Conference on Applications of Computer Vision (WACV), pp. 1–9, 2016.

[39] Damen D, Doughty H, Farinella GM, Fidler S, Furnari A, Kazakos E, Moltisanti D, Munro J, Perrett T. Will price, and michael wray. 2018. scaling egocentric vision: The epic-kitchens dataset. In: Proceedings of the European Conference on Computer Vision, pp. 753–771

[40] Oquab M, Bottou L, Laptev I, Sivic J. Learning and transferring mid-level image representations using convolutional neural networks. In: Proceedings of the IEEE Conference on Computer Vision and Pattern Recognition, pp. 1717–1724, 2014.

[41] Bianco S, Cadene R, Celona L, Napoletano P (2018). Benchmark analysis of representative deep neural network architectures. IEEE Access.

[42] Howard AG, Zhu M, Chen B, Kalenichenko D, Wang W, Weyand T, Andreetto M, Adam H. Mobilenets: efficient convolutional neural networks for mobile vision applications 2017. 10.48550/arXiv.1704.04861.

[43] Sandler M, Howard A, Zhu M, Zhmoginov A, Chen L-C. Mobilenetv2: Inverted residuals and linear bottlenecks. In: Proceedings of the IEEE Conference on Computer Vision and Pattern Recognition, pp. 4510–4520, 2018.

[44] Russakovsky O, Deng J, Su H, Krause J, Satheesh S, Ma S, Huang Z, Karpathy A, Khosla A, Bernstein M (2015). Imagenet large scale visual recognition challenge. Int J Comput Vis.

[45] Zhou B, Lapedriza A, Khosla A, Oliva A, Torralba A (2017). Places: A 10 million image database for scene recognition. IEEE Trans Pattern Anal Machine Intell.

[46] Krizhevsky A, Sutskever I, Hinton GE (2017). Imagenet classification with deep convolutional neural networks. Commun ACM.

[47] Szegedy C, Liu W, Jia Y, Sermanet P, Reed S, Anguelov D, Erhan D, Vanhoucke V, Rabinovich A. Going deeper with convolutions. In: Proceedings of the IEEE Conference on Computer Vision and Pattern Recognition, pp. 1–9, 2015.

[48] Dodge S. Karam L. Understanding how image quality affects deep neural networks. In: 2016 Eighth International Conference on Quality of Multimedia Experience (QoMEX), pp. 1–6, 2016. IEEE

[49] Vasiljevic I, Chakrabarti A, Shakhnarovich G. Examining the impact of blur on recognition by convolutional networks. 2016. 10.48550/arXiv.1611.05760.

[50] Diamond S, Sitzmann V, Julca-Aguilar F, Boyd S, Wetzstein G, Heide F (2021). Dirty pixels: Towards end-to-end image processing and perception. ACM Trans Graph (TOG).

[51] Bandini A, Dousty M, Zariffa J. A wearable vision-based system for detecting hand-object interactions in individuals with cervical spinal cord injury: First results in the home environment. In: 2020 42nd Annual International Conference of the IEEE Engineering in Medicine & Biology Society (EMBC), IEEE; pp. 2159–2162, 2020. 10.1109/EMBC44109.2020.9176274 33018434

[52] Spiers AJ, Cochran J, Resnik L, Dollar AM (2021). Quantifying prosthetic and intact limb use in upper limb amputees via egocentric video: an unsupervised, at-home study. IEEE Trans Med Robot Bionics.

[53] Abebe G, Cavallaro A. Inertial-vision: cross-domain knowledge transfer for wearable sensors. In: Proceedings of the IEEE International Conference on Computer Vision Workshops, pp. 1392–1400, 2017.

[54] Song S, Chandrasekhar V, Mandal B, Li L, Lim J-H, Sateesh Babu G, Phyo San P, Cheung N-M. Multimodal multi-stream deep learning for egocentric activity recognition. In: Proceedings of the IEEE Conference on Computer Vision and Pattern Recognition Workshops, pp. 24–31, 2016.

[55] Zhang A, Wang KC, Li B, Yang E, Dai X, Peng Y, Fei Y, Liu Y, Li JQ, Chen C (2017). Automated pixel-level pavement crack detection on 3d asphalt surfaces using a deep-learning network. Comput Aided Civ Infrastruct Eng.

