# Supplementary material for: Egocentric vision-based detection of surfaces: towards context-aware free-living digital biomarkers for gait and fall risk assessment
Source: J Neuroeng Rehabil. 2022 Jul 22;19:79. doi: 10.1186/s12984-022-01022-6 (PMC9308210; doi:10.1186/s12984-022-01022-6)
Supplement: Supplementary file 1 — Additional file 1. Preliminary results for IMU-based surface type identification. [file 12984_2022_1022_MOESM1_ESM.pdf]

Supplementary Materials (I)

A preliminary study was conducted to examine k-fold and leave-one-subject-out (LOSO) cross-validation performance of: 1) a binary IMU-based classifier to differentiate between stairs and level walking, and 2) a three-class IMU-based classifier to distinguish between three level surfaces (i.e., grass, stone, and flat/even), using an open access dataset [1] as described below. The generalizability of the terrain identification model(s) with the satisfactory LOSO performance was further assessed by conducting a case series (OAs' data) using a subset of Multimodal Ambulatory Gait and Fall Risk Assessment in the Wild (MAGFRA-W) dataset [2].

1 Material and Methods

1.1 Dataset

1.1.1 Young healthy adults

An open access dataset of 30 young healthy adults (15 males, 15 females, age = 23.5±4.2 yrs), who wore six IMUs (MTw Awinda, Xsens, Enschede, Netherlands) with a sampling frequency of 100 Hz (±160 m/s^2, ±2000 deg/s) was considered here [3]. The participants walked over nine outdoor surfaces: grade (up-, down-, and cross-slopes), level (paved, stone, grass), and stairs (material: cement, up and down), at self-selected speed. All participants walked over/through the same environment 6 times resulting in 180 trials per environment (16.4±4.2 s per trial). In the present study, the inertial data collected by a lower back-mounted IMU during 540 level walking (flat/even, stone, grass) and 360 stair negotiation (up and down) trials were considered.

While data captured from multiple IMUs or a single IMU placed on other anatomical locations (e.g., right thigh) may lead to superior terrain classification accuracies [1], considering trunk has been the most common anatomical location for IMU placement for free-living gait and fall risk assessment in the literature [4], and to achieve a less obtrusive setup for longitudinal studies, data collected from a single trunk-mounted IMU were considered for investigation.

1.1.2 OAs (for case series)

To assess the generalizability of the terrain identification models developed using young adults' data to OAs' data, a subset of MAGFRA-W dataset was taken into account. The out-of-lab data considered here were collected by a lower back-mounted IMU (Axivity, Newcastle upon-Tyne, UK; ±8 g, ±500 rad/s and 100 Hz) as well as a waist-mounted GoPro camera (GoPro Hero 5, 30fps, wide view) in public environments within Northumbria University, during which OAs navigated through different indoor and/or outdoor environments while walking alongside a researcher. The camera (providing gold/criterion standard data here) was centered at each OA's waist by means of a belt attachment to capture top-down views of feet and the regions around them. The subset considered here includes 2 OAs' data. Participant A (female, 76 yrs) and participant B (female, 76 yrs) walked over multiple surfaces.

The project received ethics approval (reference number: 17589, approval date: 4-Oct-2019) from Northumbria University Research Ethics Committee, Newcastle upon Tyne, UK. All older adults gave written informed consent before participating in the study.

1.2 Data preprocessing and feature extraction

All data were processed using MATLAB R2021. Unit conversion was performed to obtain comparable inertial data within both datasets. For each trial selected from the young adults' dataset, each of the 6 acceleration (ACC) and angular velocity ($Gyro$) signals was detrended (DC offset removal) separately. To compensate for possible orientation changes for the trunk-mounted IMU during longer data collection in the MAGFRA-W dataset, instead of detrending the full-length signals acquired for each participant, sliding windows with the length of $15s$ were applied to each signal and the overlapping data were detrended separately. Afterwards, each detrended signal in both datasets was filtered using a low-pass butterworth filter with the cut-off frequency and order of 6 Hz and 2, respectively (as suggested in [3]).

From each of the 540 level walking and 360 stair walking trials in young adults dataset, a 5-second epoch (a 6×600 matrix) was cropped from the six detrended and filtered signals and considered for feature extraction. A similar process was taken into account for the analysis of OAs' data as discussed in section 1.3.1.

Previous research showed that compliance for free-living data collection, in terms of sensor placement (location and/or orientation) may be challenging for participants. In [5], 15.6% of participants who wore accelerometers for seven days did not follow the protocol for ≥ 1 day(s) resulting in miscalculations of physical activity. Considering the goal of surface identification in free-living longitudinal studies, and since the data investigated in the present study were acquired from two sources with different data collection protocols, signal vector amplitude of the acceleration (SVA_{ACC}) and angular velocity (SVA_{Gyro}) signals, rather than all 6 axes, were employed to generally compensate for potential sensor misalignment. Thus, for each epoch, only 2 signals (i.e., SVA_{Acc}, SVA_{Gryro}) were taken into account.

The following 20 features were extracted from the SVA_{ACC} and SVA_{Gyro} components of each segment ~~(in accordance with the features considered in our previous works~~ [6; 7]): 1) range, 2) root mean square (RMS), 3) mean, 4) variance, 5) skewness, 6) kurtosis, 7) number of peaks, 8) maximum autocorrelation, 9) integral, 10) the Shannon entropy, 11) amplitude of the dominant frequency (periodogram PSD), 12) the dominant frequency in the segment, 13) maximum of signal derivative, 14) mean of the signal derivative, 15) variance of the signal derivative 16) skewness of the signal derivative, 17) kurtosis of the signal derivative, 18) RMS of the signal derivative, 19) integral of the signal derivative, and 20) the Shannon entropy of signal derivative.

The procedure discussed is this Supplementary Material for signal processing, feature extraction, and model development (see 1.3) is very similar to the proposed approach in Nouredanesh et al., 2022 [8], in which the trained IMU-based models exhibited a satisfactory performance for the detection of naturally-occurring compensatory balance reactions (missteps).

1.3 Experiments

A random forest (RF) model structure was chosen due to parallel processing and demonstrated robustness against nonlinear relationships. Here, RF models were developed to address 1) stair vs level walking differentiation (binary classification, dataset: $X_{900\times40}$ matrix), and 2) flat/even vs grass vs stone differentiation (3-class classification, dataset: $X_{540\times40}$ matrix).

Based on initial tests, an RF model with 19 trees (RF_{19}) showed satisfactory results on all validation datasets. MATLAB defaults were used for other parameters of the classifier.

For models' performance assessment, 10-fold, leave-one-trial-out (LOTO), and LOSO cross-validation measures were reported. The LOSO measures represent the average of results across all participants. For the binary classifier, samples corresponding to stairs were considered as 'positives', and the subsequent cross-validation metrics were obtained based on this consideration. For the three-class classifier, per-class accuracies were reported.

1.3.1 Validation based on OAs' data

A sliding window with the the length of 5 s and stride of 3 s was applied to the SVA_{ACC} and the corresponding SVA_{Gyro} signals attributed to two older participants. This resulted in 123 and 88 segments from participants A and B, respectively. Each segment was considered for feature extraction (as discussed in 1.2).

RF_{19} models were trained using all samples from young adult's dataset (e.g., 540 level walking and 360 stair walking samples to train the binary classifier). To indicate that the results are not impacted by the inherent model's randomness, a 'confidence score' was defined. For each segment, the average of outputs (e.g., 1: Stair, 0: Level) from 20 RF_{19}'s was defined as the segment's confidence score. Subsequently, a segment is considered as 'stair walking' if its confidence score is $\geq$0.9 (i.e., at least 18 out of 20 RF_{19} models classified the segment as stair). The centers of these segments are highlighted by circles in Fig. 1.

2 Results

Using young adults' data and LOTO cross-validation, the accuracy of 100% was achieved for the binary classifier (stair walking vs level walking). Moreover, walking patterns over flat/even, grass-, and stone-covered surfaces were detected with the LOTO accuracies of 99.44%, 100% and 99.44%, respectively, with an overall 3-class classification accuracy: 99.63%).

The 10-fold cross-validations accuracies of 99.56% and 96.11% were achieved for the binary (stair vs level walking) and three-class (flat/even vs grass vs stone) classifiers, respectively.

For the binary classifer, the mean LOSO sensitivity, specificity, and accuracy of 81.11%, 88.15%, and 85.33% were obtained, respectively. However, for the three-class classifier, the mean LOSO accuracy dropped to 40.56% (flat/even: 46.11%, stone: 33.89%, grass: 41.67%).

The unsatisfactory LOSO performance of the 3-class classifer indicates the poor generalizability of the model even to the individuals within the same population (young adults). Thus, considering the classifier's anticipated poor performance when applied to OAs' data,

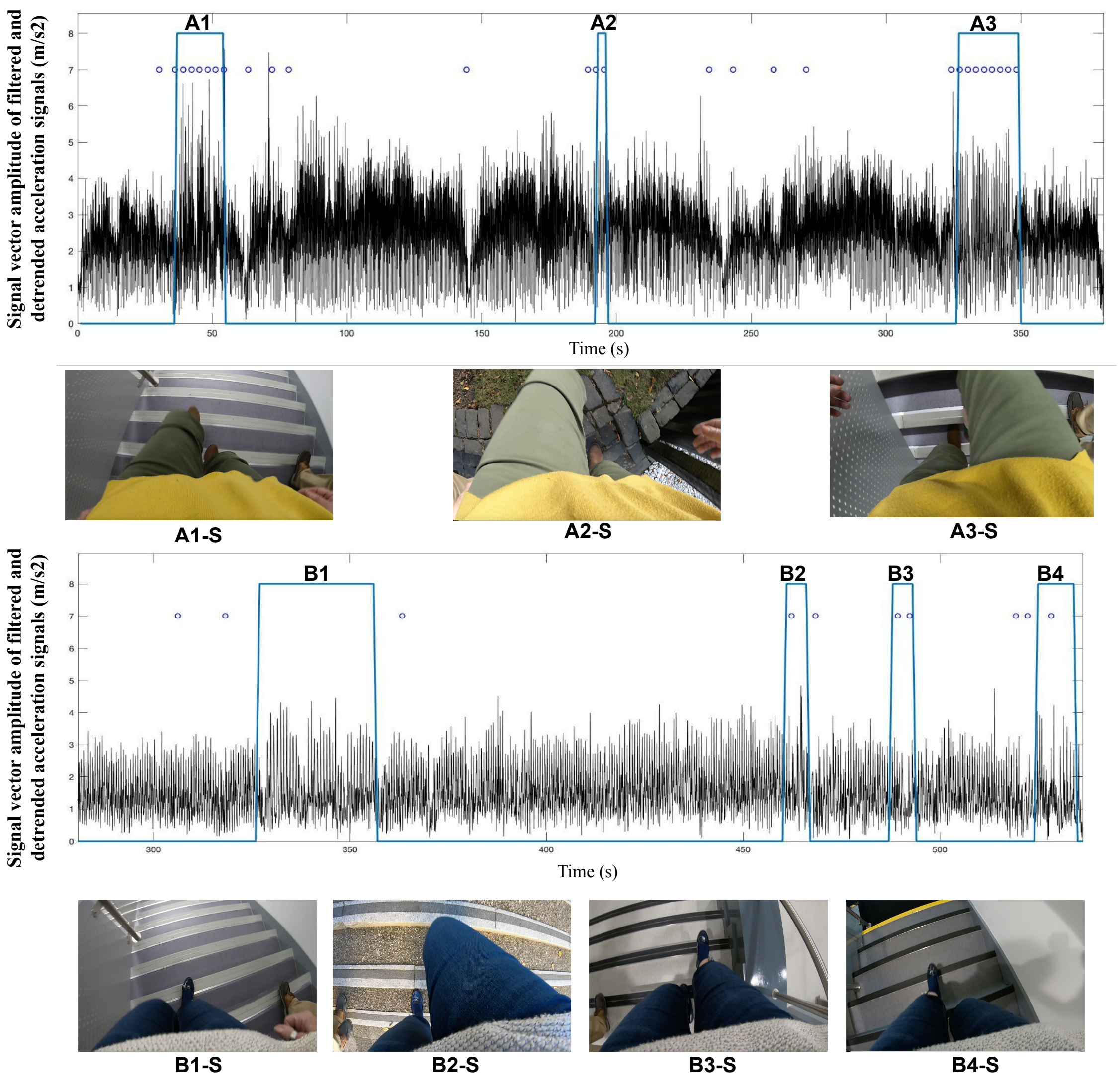

Figure 1: Participant A (female, age: 76 yrs) and participant B (female, age: 76 yrs) walked over multiple surfaces. The starts and ends of stair walking episodes were annotated (A1 to A3, and B1 to B4). The center of each 5-second sliding window with the confidence score ≥ 0.9 was highlighted by a circle. Sample (S) frames corresponding to each stair walking activity is provided under each plot.

only the stair detection model with promising performance was considered for further examination in the OA cases. To address this, the starts and ends of stair walking episodes were annotated (possible error$\approx \pm 2$ s) using the criterion standard data (sample frames as well as the annotations are provided in Fig. 1). A1 (descending), A3 (ascending) and B1 (descending) show stair walking patterns captured over the same staircase (indoor, 2 subsequent staircases, each with 10 steps, a short episode of level walking on the flooring area between the staircases was recorded). In Fig. 1, A2 (2 steps, outdoor), B2 (3 steps, outdoor), B3 (4 steps, indoor), and B4 (6 steps, indoor) show walking patterns over different indoor and outdoor steps/staircases with diverse materials (e.g., bricks).

For participant A, the stair negotiation episodes of A1, A2, and A3 were detected (the predictions with confidence scores ≥ 0.9 overlapped with the ground truth data as shown in Fig 1). For Participant B, the stair negotiation episodes were partially detected (partial overlap between the predictions and ground truth, B1 was not detected). Multiple false positives were generated for both cases (circles out of the stair negotiation annotations in Fig. 1).

3 Conclusions

Using a single trunk-mounted IMU data and random forest models, the feasibility of detecting different walking surfaces was discussed. Using young adults' data, high ($> 96\%$) 10-fold and LOTO cross-validation accuracies were obtained for both binary (stair vs gait walking) and three-class (flat/even, grass, gravel) classifiers. However, the LOSO cross-validation accuracies dropped to 85.33% and less than 50% for the binary and three-class classifiers, respectively. The considerable difference between the k-fold and LOSO cross-validation results highlights that k-fold measures may not reliably represent model's robustness against inter-participant differences that impact gait-related inertial data.

The binary classifier's satisfactory LOSO performance may be an indicator of the model's robustness against inter-participant differences (at least within the same population) suggesting that accurate differentiation between stair and level walking episodes using a trunk-mounted IMU data alone could be feasible. To further assess the generalizability of the stair walking detection model to other populations (OAs) and unseen environments (stairs with different materials and properties, e.g., riser, height and width, as well as indoor level walking surfaces) a case series was performed using 2 OAs' data, for which mixed results were achieved. On the other hand, the inferior LOSO cross-validation results for the three-class classifer (differentiation between level walking surfaces) indicated that the IMU-based models may not be robust enough against inter-participant differences associated with gait patterns. Thus, the integration of other sensor modalities may improve differentiation between different level walking surfaces.

References

[1] B. Hu, S. Li, Y. Chen, R. Kavi, and S. Coppola, "Applying deep neural networks and inertial measurement unit in recognizing irregular walking differences in the real world," *Applied Ergonomics*, vol. 96, p. 103414, 2021.

[2] M. Nouredanesh, A. Godfrey, and J. Tung, "First-person vision-based assessment of fall risks in the wild, towards fall prevention in older adults," *Journal of Computational Vision and Imaging Systems*, vol. 5, no. 1, pp. 1–1, 2019.

[3] Y. Luo, S. M. Coppola, P. C. Dixon, S. Li, J. T. Dennerlein, and B. Hu, "A database of human gait performance on irregular and uneven surfaces collected by wearable sensors," *Scientific data*, vol. 7, no. 1, pp. 1–9, 2020.

[4] M. Nouredanesh, A. Godfrey, J. Howcroft, E. D. Lemaire, and J. Tung, "Fall risk assessment in the wild: A critical examination of wearable sensor use in free-living conditions," *Gait Posture*, vol. 85, pp. 178–190, 2021. [Online]. Available: https://www.sciencedirect.com/science/article/pii/S0966636220301144

[5] M. Straczkiewicz, N. W. Glynn, and J. Harezlak, "On placement, location and orientation of wrist-worn tri-axial accelerometers during free-living measurements," *Sensors*, vol. 19, no. 9, p. 2095, 2019.

[6] M. Nouredanesh, K. Gordt, M. Schwenk, and J. Tung, "Automated detection of multidirectional compensatory balance reactions: A step towards tracking naturally occurring near falls," *IEEE transactions on neural systems and rehabilitation engineering*, vol. 28, no. 2, pp. 478–487, 2019.

[7] M. Nouredanesh and J. Tung, "Imu, semg, or their cross-correlation and temporal similarities: Which signal features detect lateral compensatory balance reactions more accurately?" *Computer methods and programs in biomedicine*, vol. 182, p. 105003, 2019.

[8] M. Nouredanesh, L. Ojeda, N. Alexander, A. Godfrey, M. Schwenk, W. Melek, and J. Tung, "Automated detection of older adults' naturally-occurring compensatory balance reactions: Translation from laboratory to free-living conditions," *IEEE Journal of Translational Engineering in Health and Medicine*, Mar. 2022.
