# Supplementary material for: Egocentric vision-based detection of surfaces: towards context-aware free-living digital biomarkers for gait and fall risk assessment
Source: J Neuroeng Rehabil. 2022 Jul 22;19:79. doi: 10.1186/s12984-022-01022-6 (PMC9308210; doi:10.1186/s12984-022-01022-6)
Supplement: Supplementary file 2 — Additional file 2. The independent training dataset curated from multiple sources. [file 12984_2022_1022_MOESM2_ESM.pdf]

Supplementary Materials (II)

Table 1 Training dataset, the relevant images, mostly from top-down view, were either extracted from available datasets, MINC-2500, GTOS and EgoSeg, or collected by the authors.

	Total	MINC-2500	GTOS(-Mobile)	EgoSeg	Author
**Outdoor**					
Pavement (Asphalt /Brick/Cement)	1503	250 (brick)	591 (brick+ asphalt+ cement+ stone asphalt + stone cement)	662	
Foliage/Grass	1382	0	1227 (turf+leaf+grass)	155	0
Gravel/Stone	1266	0	1266 (pebble+shale)	0	0
Snow	323	0	0	0	323
Soil	1299		1230	69	0
**Indoor**					
High-friction materials	2184 (e.g., fabric and carpet (top-down view))	2184	-	-	-
Wood (top-down view)	445	445	-	-	-
Indoor tiles (top-down view)	1018	810	-	208	-

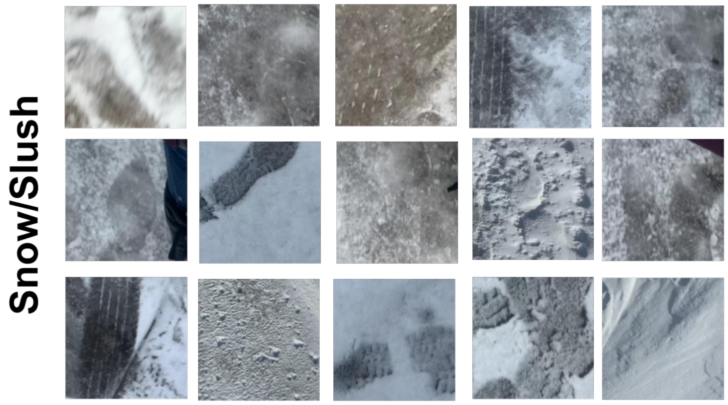

Figure 1 Sample patches representing class 'slippery/snow', the data was captured from a smartphone from waist level.
