# Supplementary material for: Egocentric vision-based detection of surfaces: towards context-aware free-living digital biomarkers for gait and fall risk assessment
Source: J Neuroeng Rehabil. 2022 Jul 22;19:79. doi: 10.1186/s12984-022-01022-6 (PMC9308210; doi:10.1186/s12984-022-01022-6)
Supplement: Supplementary file 3 — Additional file 3. Preliminary terrain type identification results using MINC-2500 and GTOS datasets. [file 12984_2022_1022_MOESM3_ESM.pdf]

# Supplementary Materials (III)

Table 2: For *EgoTerrainNet*-Outdoor, MobileNetV2 was fine-tuned on images only from GTOS(-mobile) relevant classes with a relatively balanced distribution (asphalt/cement/stone-asphalt: 1309, leaf/grass/dry leaf/turf: 1226, Soil: 1230, pebble/Shale: 1266). For *EgoTerrainNet*-Indoor, all 2,500 images in 'Wood', 'Carpet' and 'Tiles' from MINC dataset were used to fine-tune the MobileNetV2. The validation accuracies of 99.20 and 87.56 for the outdoor and indoor versions were obtained, respectively. For the indoor version. Confusion Matrices representing test results on MAGFRA-W datasets.

| | | Outdoor | | | | | | | Indoor | | | | | |
|---|---|---|---|---|---|---|---|---|---|---|---|---|---|---|
| | | Pavement | Foliage | Gravel | Soil | Snow | $Num$ | Acc | | HFM | Tile | Wood | $Num$ | Accuracy |
| $OA_1$ | Pavement | 4161 | 1 | 19 | 920 | 0 | 5101 | 81.57 | HFM | 568 | 161 | 1349 | 2078 | 27.33 |
| | Foliage | 89 | 11 | 0 | 53 | 0 | 153 | 7.18 | Tile | 12 | 402 | 30 | 444 | 90.54 |
| | Gravel | 0 | 0 | 0 | 0 | 0 | 0 | 0 | Wood | 0 | 0 | 0 | 0 | - |
| | Soil | 20 | 4 | 0 | 15 | 0 | 39 | 38.46 | | | | | | |
| $OA_2$ | Pavement | - | - | - | - | - | - | - | HFM | 98 | 1 | 7 | 106 | 92.45 |
| | Foliage | - | - | - | - | - | - | - | Tiles | 0 | 0 | 0 | 0 | - |
| | Gravel | - | - | - | - | - | - | - | Wood | 0 | 0 | 0 | 0 | - |
| | Soil | - | - | - | - | - | - | - | | | | | | |
| $OA_3$ | Pavement | 47 | 3 | 1 | 0 | 0 | 51 | 92.16 | HFM | 48 | 2 | 10 | 60 | 80 |
| | Foliage | 1 | 7 | 0 | 0 | 0 | 8 | 87.50 | Tiles | 13 | 1 | 2 | 16 | 6.25 |
| | Gravel | 0 | 0 | 0 | 0 | 0 | 0 | 0 | Wood | 0 | 0 | 0 | 0 | - |
| | Soil | 3 | 0 | 0 | 0 | 0 | 3 | 0 | | | | | | |
| $OA_4$ | Pavement | 1671 | 2 | 6 | 1672 | 0 | 3351 | 49.86 | HFM | 788 | 223 | 663 | 1674 | 47.07 |
| | Foliage | 14 | 23 | 0 | 8 | 0 | 45 | 51.11 | Tiles | 1 | 584 | 26 | 611 | 95.58 |
| | Gravel | 0 | 0 | 0 | 0 | 0 | 0 | 0 | Wood | 0 | 0 | 0 | 0 | - |
| | Soil | 0 | 0 | 0 | 0 | 0 | 0 | 0 | | | | | | |
| $OA_5$ | Pavement | 1592 | 9 | 14 | 330 | 0 | 1945 | 81.85 | HFM | 1037 | 30 | 456 | 1523 | 68.08 |
| | Foliage | 28 | 51 | 0 | 28 | 0 | 107 | 47.66 | Tiles | 28 | 295 | 20 | 343 | 86.00 |
| | Gravel | 12 | 6 | 240 | 0 | 0 | 258 | 93.02 | Wood | 0 | 0 | 0 | 0 | - |
| | Soil | 0 | 0 | 0 | 0 | 0 | 0 | 0 | | | | | | |
| $OA_6$ | Pavement | 74 | 2 | 2 | 0 | 0 | 78 | 94.87 | HFM | 234 | 3 | 24 | 261 | 89.65 |
| | Foliage | 21 | 8 | 0 | 0 | 0 | 29 | 27.58 | Tiles | 76 | 132 | 33 | 241 | 54.77 |
| | Gravel | 0 | 0 | 0 | 0 | 0 | 0 | 0 | Wood | 0 | 0 | 0 | 0 | - |
| | Soil | 0 | 0 | 0 | 0 | 0 | 0 | 0 | | | | | | |
| $OA_7$ | Pavement | 1804 | 54 | 36 | 159 | 0 | 2053 | 87.87 | HFM | 455 | 91 | 533 | 1079 | 42.16 |
| | Foliage | 74 | 61 | 0 | 24 | 0 | 159 | 38.36 | Tiles | 6 | 212 | 8 | 226 | 93.80 |
| | Gravel | 33 | 9 | 309 | 0 | 0 | 351 | 88.03 | Wood | 0 | 2 | 45 | 47 | 95.74 |
| | Soil | 0 | 0 | 0 | 0 | 0 | 0 | 0 | | | | | | |

| | | | | | | | | | | | | | | |
|---|---|---|---|---|---|---|---|---|---|---|---|---|---|---|
| $P_8$ | Pavement | 4956 | 200 | 0 | 1083 | 0 | 6239 | 79.43 | HFM | 243 | 93 | 91 | 427 | 56.90 |
| | Foliage | 12 | 139 | 0 | 0 | 0 | 151 | 92.05 | Tiles | 0 | 0 | 0 | 0 | 0 |
| | Gravel | 0 | 0 | 0 | 0 | 0 | 0 | 0 | Wood | 83 | 51 | 178 | 312 | 57.05 |
| | Soil | 0 | 0 | 0 | 0 | 0 | 0 | 0 | | | | | | |
| $OA_9$ | Pavement | - | - | - | - | - | - | - | HFM | 604 | 130 | 253 | 987 | 61.19 |
| | Foliage | - | - | - | - | - | - | - | Tiles | 2 | 342 | 7 | 351 | 97.43 |
| | Gravel | - | - | - | - | - | - | - | Wood | 0 | 0 | 0 | 0 | - |
| | Soil | - | - | - | - | - | - | - | | | | | | |
